# Algicidal Effects of Prodigiosin on the Harmful Algae *Phaeocystis globosa*

**DOI:** 10.3389/fmicb.2016.00602

**Published:** 2016-04-26

**Authors:** Huajun Zhang, Yun Peng, Su Zhang, Guanjing Cai, Yi Li, Xujun Yang, Ke Yang, Zhangran Chen, Jun Zhang, Hui Wang, Tianling Zheng, Wei Zheng

**Affiliations:** ^1^Key Laboratory of the Ministry of Education for Coastal and Wetland Ecosystems, School of Life Sciences, Xiamen UniversityXiamen, China; ^2^School of Marine Sciences, Ningbo UniversityNingbo, China; ^3^Department of Biology, College of Science, Shantou UniversityShantou, China

**Keywords:** red tide, *Phaeocystis globosa*, algicidal bacterium, prodigiosin, anti-algal

## Abstract

*Phaeocystis globosa* blooms can have negative effects on higher trophic levels in the marine ecosystem and consequently influence human activities. Strain KA22, identified as the bacterium *Hahella*, was isolated from coastal surface water and used to control *P. globosa* growth. A methanol extract from the bacterial cells showed strong algicidal activity. After purification, the compound showed a similar structure to prodigiosin when identified with Q-Exactive Orbitrap MS and nuclear magnetic resonance spectra. The compound showed algicidal activity against *P. globosa* with a 50% Lethal Dose (LD_50_) of 2.24 μg/mL. The prodigiosin was stable under heat and acid environment, and it could be degraded under alkaline environment and natural light condition. The growth rates of strain KA22 was fast in 2216E medium and the content of prodigiosin in this medium was more than 70 μg/mL after 16 h incubation. The compound showed particularly strong algicidal activity against *Prorocentrum donghaiense, P. Globosa*, and *Heterosigma akashiwo*, but having little effect on three other phytoplankton species tested. The results of our research could increase our knowledge on harmful algal bloom control compound and lead to further study on the mechanisms of the lysis effect on harmful algae.

## Introduction

As one of the most serious results of eutrophication, HABs in coastal waters cause irreversible problems with regard to effective utilization of water resources in fisheries, water supply (Anderson, 1997; Heisler et al., 2008; Anderson et al., 2012). The euryhaline genus *Phaeocystis* is one of the most widespread marine haptophytes, with species sharing the ability to produce nearly monospecific blooms of gelatinous colonies in coastal waters (Schoemann et al., 2005). HABs dominated by *Phaeocystis globosa* are recurrent events in marine ecosystems (Lamy et al., 2009), and termination of these blooms can cause excessive foam, which becomes a nuisance for socio-economical activities (Baudoux and Brussaard, 2005).

This has led to heightened scientific and regulatory attention, and the development of many new technologies and approaches for research and management. In order to terminate HABs, several approaches including physical and chemical methods have been carried out (Moss et al., 1977; Pierce et al., 2004; Sengco and Anderson, 2004). Biological methods have received particular interest in recent years because of their potential effectiveness, species specificity, and ecofriendly nature (Anderson, 2009; Zheng et al., 2012). Biologically active compounds, produced by bacteria particularly the algal-toxins, are one of the most interest choice. Most reported algicidal bacteria exert algicidal activity by excreting extracellular substances, a process known as allelopathy (Mayali and Azam, 2004; Kim et al., 2009). The discovery of allelopathy indicated that the natural allelochemicals produced by autotrophs might provide the potential algal growth inhibitors to control HABs that meet these requirements. However, traditional approaches to study allelochemicals have mainly focused on the isolation of allelopathic compounds from aquatic macrophytes and terrestrial plants (Ball et al., 2001; Tsuda et al., 2005; Ni et al., 2012), while few studies have focused on the potential isolation of algicide from marine related bacteria.

In this study, our essential tasks included (1) isolation and characterization of the algicidal compound released from the bacterium KA22, (2) revealing the character of this algicidal compound, and (3) exploring the mechanism of the action of the compound.

## Materials and Methods

### Algal Cultures

Cultures of the experimental alga, *P. globosa*, were supplied by the State Key Laboratory of Marine Environmental Science (Xiamen University). The axenic culture of the algal was obtained according to the reported methods (Su et al., 2007a). The cultures were incubated in sterile f/2 medium (without silicate) prepared with natural seawater (Guillard, 1975) at 20 ± 1°C under a 12:12 h light–dark cycle with a light intensity of 50 μmol photons m^–2^ s^–1^. Exponential phase axenic cultures (about 4 × 10^6^ cells/mL) were used for all experiments.

### Identification of the Algicidal Microorganism KA22

Water samples were collected from coastal surface water of Xiamen (24°25′N, 118°06′E), when the biomass of *P. globosa* was very high from July to August 2012. Samples were serially diluted with sterile seawater, and 0.1 mL aliquots of each dilution were spread onto 2216E (peptone 5 g/L, yeast extract 1 g/L, ferric phosphorous acid 0.1 g/L, dissolved in natural seawater, pH7.6–7.8) agar plates followed by incubation for 3 days at 28°C. Colonies with distinct morphologies were further purified several times until individual colonies were obtained. Then the isolates were inoculated into 2216E broth followed by incubation for 3 days and 150 rpm at 28°C to test their algicidal effect. After that, a strain named KA22 was characterized by the 16S rRNA gene according to the reported methods (Su et al., 2007b). A phylogenetic tree was constructed using MEGA 5.0 software and using neighbor-joining analysis (Pandey et al., 2013). Cell morphology was observed using transmission electron microcopy (JEM-2100HC, Japan).

### Purification and Identification of the Anti-algal Compound

Supplementary Figure S1 is a flow diagram of the anti-algal compound purification procedure. Bacterial cells were separated from 2216E broth after 3 days cultivation. After that, cells were extracted with methanol and the supernatant was concentrated followed by extraction with methanol for 2 h. Then the methanol fractions were extracted using ethyl acetate in order to remove the insoluble fraction. The crude extracts dissolved in ethyl acetate were applied to a Sephadex LH-20 (Amersham Biosciences) column with 100% methanol as eluent. After rotary evaporation, three fractions were dissolved in DMSO then added into algal culture to detect their anti-algal effect. The most active fraction was eluted using size gel exclusion chromatography and then subjected to silica gel column chromatography (170 mm × 30 mm in dimension and with a silica particle size of 200–300 mesh) and eluted with hexane-ethyl acetate (20:1, 10:1, 8:1, 6:1, 5:1, 3:1, 0:1). Fractions that exhibited algicidal activity were reloaded on the silica gel column chromatography system (170 × 30 mm in dimension and with a silica particle size of 500 mesh) and again eluted with the same volume of hexane-ethyl acetate. Finally, the purified compound was dissolved in ethyl acetate. The NMR spectra of the compound were recorded in CDCl_3_ using a DRX500 instrument (Bruker Biospin, Co., Karlsruhe, Germany) at 25°C. Trimethylsilyl (TMS) was used as an internal standard. The active fraction was also dissolved in acetonitrile and analyzed using Q-Exactive Orbitrap MS.

### Properties of the Anti-algal Compound

The purified compound was first to analyzed with full wave scanning to assay its absorbance length. The purified compound was dissolved in methanol with the concentration of 34 μg/mL to study its properties. The compound was incubated at 40, 60, and 80°C for 3 h to detect its heat stability and room temperature served as the control. To investigate the effect of pH on the stability of the compound, the pH of the solution was adjusted to 3, 4, 5, 8, 9, and 10 using 0.1 M HCl and 0.1 M NaOH. The solution was also exposed to natural light for 8 h to study its light stability. The purified compound without any treatment served as control. After this, all groups were analyzed with full wave scanning and absorbance.

### Anti-algal Activity of the Isolated Compound

The purified compound was dissolved in DMSO to test its algicidal effect on *P. globosa.* The compound were added into the algal cultures in final concentrations of 1, 3, 5, and 10 μg/mL, respectively. DMSO was also added into algal culture in the same volume serving as control. The algal growth was monitored with algal cell fluorescence (aaaex = 440 nm, aaaem = 680 nm) in 24, 48, and 72 h. The algicidal activity in this study was calculated using the following formula:

Algicidal activity (%)=(Fc−Ft)/Fc×100%

While Fc and Ft represent the fluorescent intensity of normal and treatment algal cells. The determination of LD_50_ was calculated using SPSS (version 19.0) after treatment with the concentration of 1, 3, 5, and 10 μg/mL for 24 h.

The effects of the anti-algal compound on the lysis of *P. globosa* cells throughout the algicidal process were observed microscopically (Olympus BX41, Chiyoda-ku, Tokyo, Japan). Photographs of algal cell morphology were taken with a DP50 digital camera (Olympus; Wang et al., 2010).

### Relationship of Bacterial Growth Rate and Algicidal Compound Yield

The bacterial was first cultured in 2216E broth until its OD_600_ was about 0.7 then 1 mL of this broth was inoculated into 100mL fresh 2216E culture followed by incubation at 28°C. After 4, 8, 12, 16, 20, 24, 28, and 32 h culture, 0.5 mL bacterial culture was taken to assay its OD_600_ and another 0.5 mL was used to detect the yield of algicidal compound after extracted with methanol. Then we detected the absorbance of the extract in 535 nm.

### Algicidal Range of Algicidal Compound

To investigate the algicidal specificity of this compound, the following HABs species were chose: *Prorocentrum donghaiense, Alexandrium tamarense, Heterosigma akashiwo, Skeletonema costatum, Scrippsiella trochoidea.* Two milliliter of these algal were cultured in microwell plate then algicidal compound was added into each algal species culture at the concentration of 5 μg/mL. DMSO served as control was also added into the culture at the same volume. After 3 days treatment, the algicidal activity was calculated as described above.

### Statistical Analysis

All data were presented as means ± standard deviation of the mean (three replicates) and were evaluated using one-way analysis of variance followed by the least significant difference test, with *p* < 0.01 and *p* < 0.05 (Origin 8.5 for Windows).

## Results

### Characterization and Identification of Strain KA22

**FIGURE 1:**
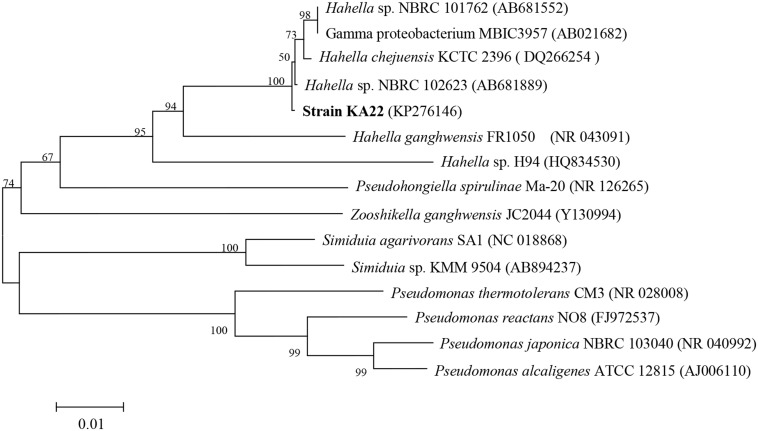
**Phylogenetic tree based on 16S rDNA sequences showing the position of strain KA22, and representatives of *Hahella*.** The scale bar represents 0.01 substitutions per nucleotide position.

PCR amplification of the 16S rRNA gene together with sequencing showed that KA22 (GenBank accession number: KP276146) had the most similarity (98.68%) with *Hahella chejuensis*, and the genetic distance was the smallest (**Figure 1**). Transmission electron microscopy (TEM) analysis showed that it was rod shaped (about 8.5 μm × 0.5 μm; Supplementary Figure S2).

### Identification of Anti-algal Compound

**FIGURE 2:**
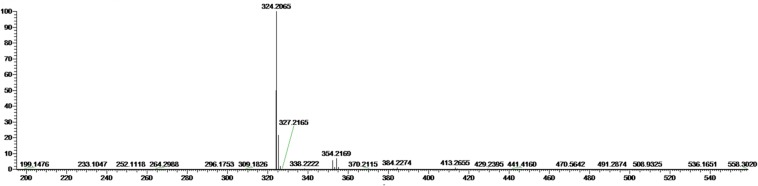
**Mass spectrum of the algicidal compound from Q-Exactive Orbitrap MS.** The molecular weight of prodigiosin is 323.2 (m/z 324.2 = M_r_ + H^+^).

**FIGURE 3:**
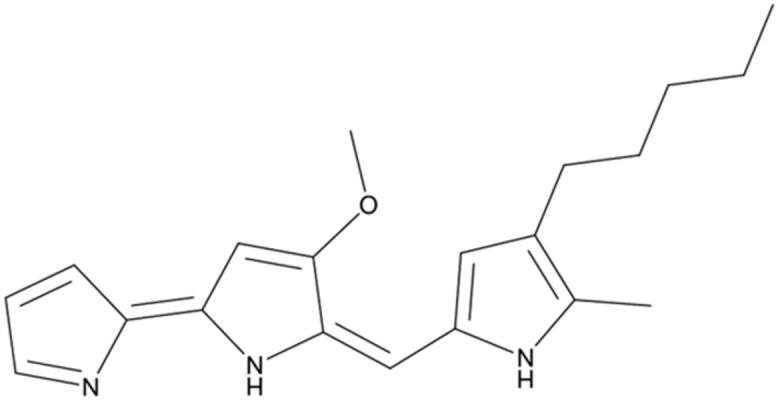
Structure of prodigiosin.

The most active fraction was obtained from KA22 culture and it was identified using Q-Exactive Orbitrap MS and NMR. The algicidal compound was obtained as a red crystal and the molecular formula was determined to be C_20_H_26_ON_3_ using the Q Exactive Q-Exactive Orbitrap MS data (*m/z* 324.20 for [M+H]^+^; **Figure 2**), which was also confirmed by the NMR results. The ^13^C-NMR resulted in the raw data of 58.5, 14.1, 10.5, 31.8, 30.3 25.6, 22.6, 122.5, 120.8, 115.8, 112.2, 109.8, 95.4, 169.0, 159.4, 138.3, 136.4 128.7, 127.5, 124.1 (See Supplementary Figures S3 and S4; **Table 1**). The^1^H-NMR spectrum yielded the following results: δ(H) = 6.83 (s), 6.69 (d, *J* = 2.56 Hz), 6.66 (s), 6.36 (s), 6.16 (s), 6.09 (s), 3.99 (s), 2.24 (t, *J* = 7.57 Hz), 1.78 (s), 1.47 (m), 1.32 (m), 1.28 (m), 0.88 (t, *J* = 6.92; Supplementary Figure S5). A preliminary structure of this algicidal compound can be built up including other spectra (Supplementary Figures S6–S8) and the structure of the compound derived from the above analysis (prodigiosin) is shown in **Figure 3** with molecular weight of 323.2.

**Table 1:** ^1^H and ^13^C-NMR data obtained in CDCl_3_ at 600 MHz for the algicidal compound.

Position	DEPT	δ(C)	δ(H)
1	N		
2	CH	122.5	6.66(s)
3	CH	112.2	6.69(d, 2.56 Hz)
4	CH	109.8	6.16(s)
5	C	128.7	
6	C	159.4	
7	CH	95.4	6.09(s)
8	C	169.0	
9	C	138.3	
10	CH	115.8	6.83(s)
11	C	127.5	
12	CH	120.8	6.36(s)
13	C	124.1	
14	C	136.4	
15	CH_3_	10.5	1.78(s)
16	CH_2_	25.6	2.24(t, 7.57 Hz)
17	CH_2_	30.3	1.47(m)
18	CH_2_	31.8	1.28(m)
19	CH_2_	22.6	1.32(m)
20	CH_3_	14.1	0.88(t, 6.92)
21	CH_3_	58.5	3.99(s)
22	NH		
23	NH		

### The Properties of Prodigiosin

**FIGURE 4:**
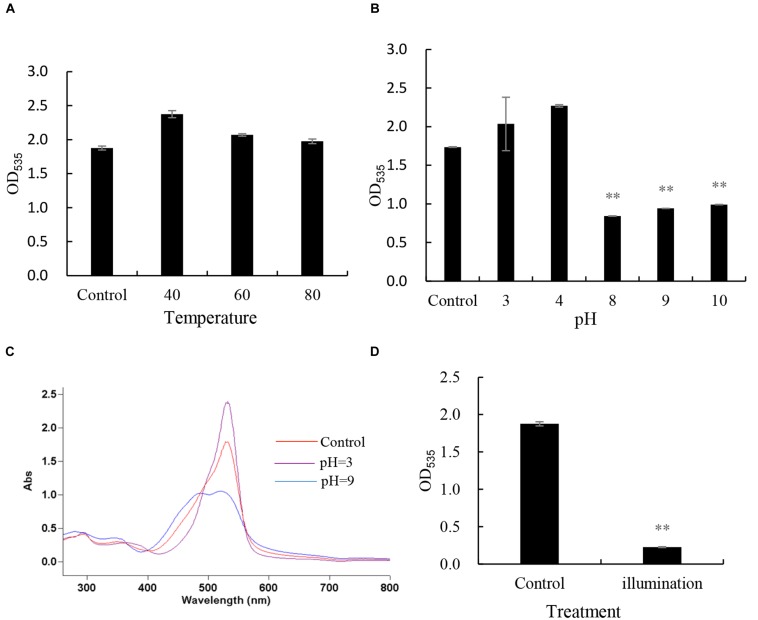
**The stability of prodigiosin under different conditions. (A)** heat stability, prodigiosin was treated by different temperature(40, 60, 80°C); **(B,C)** pH stability, prodigiosin was treated under different pH condition(3, 4, 8, 9, and 10); **(D)** light stability, prodigiosin was illuminated in natural light for 4 h. All data were mean ± SD (*n* = 3). ^∗∗^represents statistical significance at *p* < 0.01.

The stability of prodigiosin was tested under different heat, pH and natural light. When we detected the absorbance under 535 nm, it remained stable after incubation at 40, 60, and 80°C for 3 h (**Figure 4A**). However, when the prodigiosin was treated with different pH levels (**Figure 4B**), the OD_535_ value showed a great change when the pH was adjusted to 5, 8, 9, and 10 compared with the control and similar results were obtained from full wave scanning (**Figure 4C**). The prodigiosin was not sensitive to pH less than 5. The prodigiosin was also very unstable when it was illuminated under natural light for 4 h and the OD_535_ value decreased significantly (*p* < 0.01) when compared to the control (**Figure 4D**).

### Anti-algal Activity of the Isolated Prodigiosin

**FIGURE 5:**
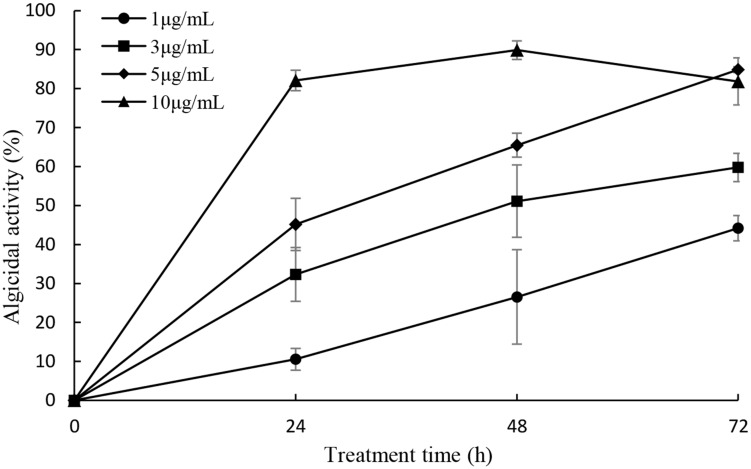
**Algicidal activity of prodigiosin on *P. globosa* at the concentration of 1, 3, 5, and 10 μg/mL.** The algicidal activity was detected at the time point of 24, 48, and 72 h. All data were mean ± SD (*n* = 3).

In order to detect the algicidal effect of the prodigiosin, it was added into *P. globosa* cultures in the concentration of 1, 3, 5, and 10 μg/mL (**Figure 5**). The concentration of 1 μg/mL showed lower alga-lytic activity compared with other three groups and algicidal activity increased as treatment time prolonged. When the prodigiosin concentration reached 3 μg/mL, the algicidal activity was about 60% at 72 h treatment and it was about 84% when the concentration was increased to 5 μg/mL. The algicidal activity reached 82% at 24 h when treated with 10 μg/mL prodigiosin and it remained the same until 72 h. Based on the algicidal effect and the dose of prodigiosin, 5 μg/mL was a better concentration for further study because it could reach high algicidal activity with fewer dose. And the compound showed algicidal activity against *P. globosa* with a LD_50_ of 2.24 μg/mL in 24 h.

**FIGURE 6:**
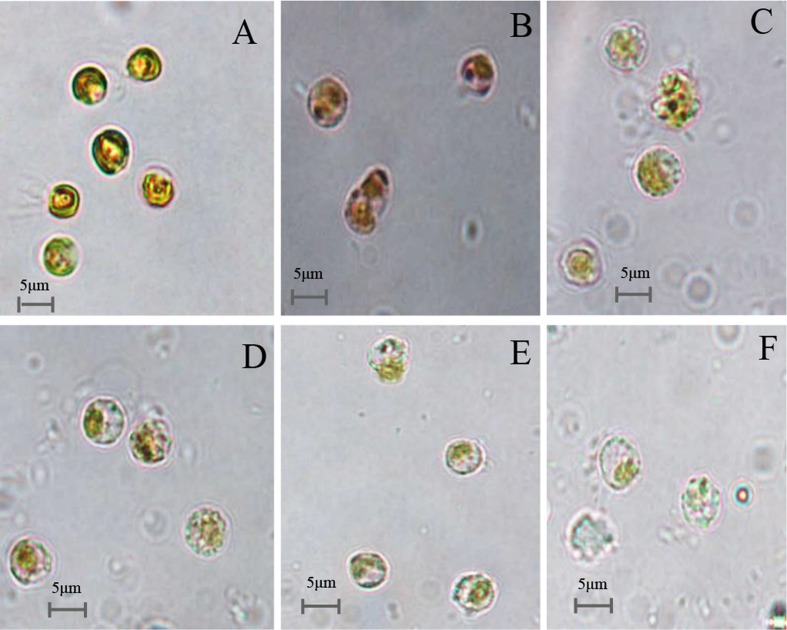
**Microscopy images of the cellular morphology of prodigiosin-treated *P. globose* cells. (A)** Control cells; **(B–F)** 12, 24, 36, 48, and 72 h treatment. Scale bars = 5 μm.

The effects of prodigiosin on the lysis of *P. globosa* cells throughout the algicidal process were observed under microscopy and shown in **Figure 6**. Normal *P. globosa* cells were shown in **Figure 6A**. In the presence of prodigiosin, cell shrinkage and plasmolysis were observed and the cytoplasm showed condensation (**Figure 6B**) compared with normal cells. As exposure time increased, algal cells lysed with membrane rupture which resulted in cellular components release (**Figures 6C–E**), and cells disintegration finally (**Figure 6F**). Moreover, many algal cells with flagella were observed in normal cells, but they lost their flagella after prodigiosin treatment and this indicated that cells lost their motility.

### Relationship of Bacterial Growth Rate and Algicidal Compound Yield

**FIGURE 7:**
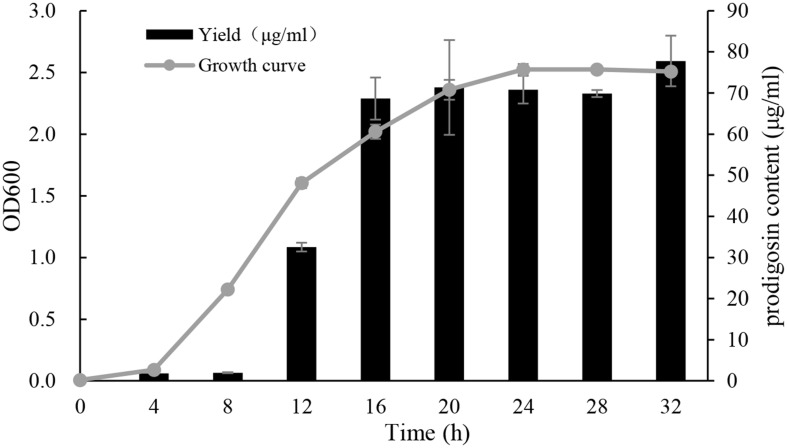
**Growth curve of KA22 at optimal culture condition (28°C, 150 rpm) and the yield of algicidal compound.** The test was carried out at the time point of 0, 4, 8, 12, 16, 20, 28, and 32 h.

The growth curve and algicidal compound yield of strain KA22 were inspected every 4 h, over a 32 h period (**Figure 7**). It showed that the yield of algicidal compound was bacterial growth-dependent, since the yield of algicidal compound increased during exponential phase and kept steady during stationary phase. After 16 h culture, the content of algicidal compound was more than 70 μg/mL, and the highest content was 77.8 μg/mL at 32 h.

### Algicidal Range of the Compound

**FIGURE 8:**
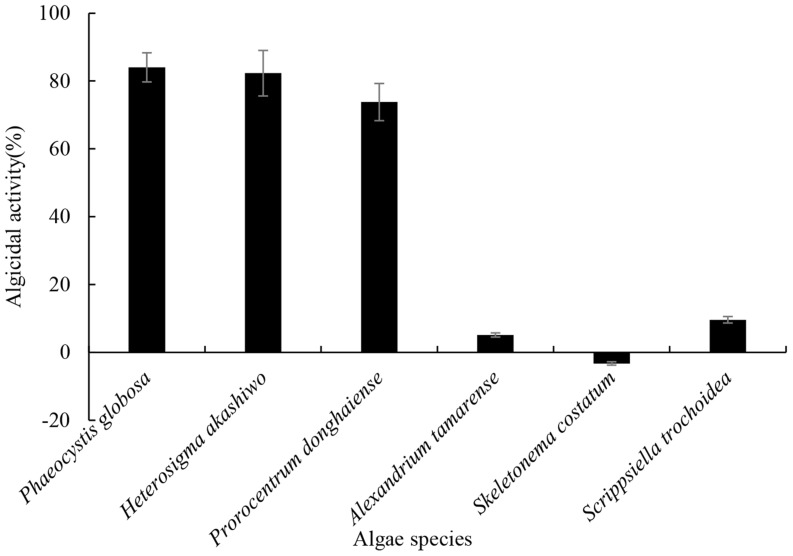
**The algicidal range of prodigiosin against other HABs species.** The 5 μg/mL prodigiosin was added into each algal culture. Algicidal activity was calculated after 72 h and DMSO served as control. All data were mean ± SD (*n* = 3).

The algicidal range of this compound against other HAB species is presented in **Figure 8**. As shown in **Figure 8**, the algicidal activities against HABs were as follows: *P. globosa* (84.0%), *H. akashiwo* (82.3%), *P. donghaiense* (73.8%), *A. tamarense* (5.1%), *S. costatum* (-3.3%) and *S. trochoidea* (9.6%). The compound showed particularly strong algicidal activity against *P. globosa*, *H. akashiwo*, *P. donghaiense*. However, it is interesting that the compound did not show algicidal activity against the other two dinoflagellate species.

## Discussion

Bacteria and phytoplankton dynamics are closely linked in coastal marine environments, with correlations frequently observed between bacterial and phytoplankton biomass (Fuhrman et al., 1980). Many studies have shown interactions between phytoplankton and bacterial communities but little is known about how these communities interact with each other at the species level (Bratbak and Thingstad, 1985; Mayali and Azam, 2004; Rooney-Varga et al., 2005). Several lines of evidence on HABs has revealed that marine bacteria are capable of promoting or inhibiting phytoplankton growth (Fukami et al., 1997; Iwata et al., 2004; Rooney-Varga et al., 2005). Some studies revealed that bacteria communities during algal blooms in marine waters are mainly dominated by *Alphaproteobacteria*, *Gammaproteobacteria*, and *Bacteroidetes* (Brussaard et al., 2005; Teeling et al., 2012). The killing of phytoplankton by bacteria such as KA22 may be a significant factor influencing the population dynamics of phytoplankton in marine environment and may contribute to the sudden disappearance of HABs in the coastal sea. Bacterial destruction of phytoplankton may also be a factor that regulates primary productivity in marine ecosystems.

In this study, the bacteria KA22 exhibited high algicidal activity against *P. globosa.* It belonged to the *Hahella* sp. and shared the highest similarity with *Hahella chejuensis* (Lee et al., 2001; Jeong et al., 2005) (**Figure 1**). *Hahella* sp. is a culturable member of the oceanic *Gammaproteobacteria*, which is one of the most prevalent prokaryotic groups present in marine environments. Based on our investigation (unpublished data), *Gammaproteobacteria* is the dominant bacterial species in Xiamen sea during HABs period in the past 3 years, suggesting that they may affect algal bloom dynamics. Therefore, this isolate may be involved in the regulation of algal blooms as reported for other algicidal bacteria (Hare et al., 2005; Kim et al., 2008). Moreover, it is much easier to obtain algicidal bacteria from the phycosphere of algae.

Bacterial interactions with HAB species have been reviewed by Doucette et al. (1998). In general, bacteria exhibit algicidal activity mainly through direct or indirect attack. Indirect attacks are thought to be chemically mediated and these chemicals are allelochemicals. The reported anti-algal allelochemicals in the literature include mainly fatty acids, polyphenols, terpenoids, and polyether (Macías et al., 2008). But few algicidal compounds have been reported to lyse *P. globosa* and our research could expand the knowledge of this incomplete field. After extraction of the red pigment of KA22, we found that the red prodigiosin-like pigment was responsible for the rapid cell lysis of *P. globosa* (**Figures 2** and **3**; Supplementary Figures S3–S8; **Table 1**) but there are some differences with the reported structure (Jeong et al., 2005), such as the site of double bond in three pyrrole rings. Prodigiosin isolated from KA22 in the present study showed strong growth inhibition on harmful algae *P. globosa* with a LD_50_ of 2.24 μg/mL (**Figures 5** and **6**). *P. globosa* cells have flagella in normal cells, but cells cannot swim around when observed by microscopy after prodigiosin treatment. The results indicated that they lost their flagella which could not be observed by microscopy. Some algicidal compounds showed similar algicidal effect on harmful algae with prodigiosin. Alamsjah et al. (2005) had reported some polyunsaturated fatty acids showing algicidal activity against *Heterosigma akashiwo* with a LC_50_ of 1.35 μg/mL. Previous studies found that *Myriophyllum spicatum* released four polyphenols and fatty acids that exhibited inhibition effects on algae (Nakai et al., 2005). Three diterpenes isolated from brown algae *Dictyota dichotoma* showed algicidal activity against red-tide phytoplanktons (Kim et al., 2006). And our results indicated that prodigiosin is an effective algicidal compound against *P. globosa.* Moreover, prodigiosin is degradable under natural light (**Figure 4D**) which poses a huge advantage for its potential use as an HAB controller because it would be degraded automatically in the environment. But it is also important to study the light degradation product of prodigiosin in future.

The testing of algicidal activity against *P. globosa* was shown in **Figure 5** and it seemed that the algicidal activity of prodigiosin was dose-dependent, since the algicidal activity increased as the concentration of prodigiosin increased. It has been reported that the algicidal activity can increase as the bacterial cell density increased (Nakashima et al., 2006; Mu et al., 2007), because it can produce more secondary metabolites during growth phase. Bacteria exert algicidal activity mainly through two ways: direct or indirect attack (Mayali and Azam, 2004). Algicidal bacteria such as strain KA22 can release dissolved algicide to kill target HABs and it belonged to indirect way. **Figure 6** showed that prodigiosin could exhibit high algicidal activity without the direct contact with the target algal cells. Algicidal activity through released algicides clearly provides a competitive benefit to this segment of the microbial community (Mayali and Azam, 2004; Roth et al., 2008; Kim et al., 2009). Current mitigation strategies for HABs in natural environments such as the application of clay flocculants or chemical surfactants can have negative long-term effects on higher trophic levels (Sengco and Anderson, 2004; Jeong et al., 2008; Su et al., 2011). Nevertheless, algicidal compound from bacteria may have little effect on non-target algal classes, fish or aquatic life in general. The algicide produced by strain KA22 targets a relative narrow range of HABs while having little to no effect on three other phytoplankton species tested (**Figure 8**). And this information suggests that prodigiosin is a potential algicide and its application may be high-efficiency and relative safety.

Strain KA22 was associated with the population dynamics of phytoplankton in natural marine environments and may contribute to the sudden disappearance of HABs in the coastal sea. It secretes a bioactive compound that exhibit high algicidal activity when introduced into *P. globosa* cultures. The bioactive compound was then identified as prodigiosin and it can be degraded automatically in the environment. Moreover, the algicide had a relative narrow algicidal range which suggests that its application may be safe. Current research is focused on the property of prodigiosin, and further studies are essential before this algicide can be practically applied to the regulation of red tide occurrences.

## Author Contributions

HZ contribute for conception and design, drafting of the article, technical and logistic support, analysis and interpretation of the data. YP and SZ contribute for collection and assembly data and analysis the data. GC, YL, XY, KY, and ZC contribute for statistical expertise and collection and assembly data. JZ, HW contribute for critical revision of the article for important intellectual content. WZ contribute for obtaining of funding and provision of study materials. TZ contribute for obtaining of funding and final approval of the article. All authors had reviewed the manuscript.

## Conflict of Interest Statement

The authors declare that the research was conducted in the absence of any commercial or financial relationships that could be construed as a potential conflict of interest.

## Abbreviations

CAT: catalase
DAPI: 4′,6-diamidino-2-phenylindole
DMSO: dimethyl sulfoxide
GPx: glutathione peroxide
GR: glutathione reductase
HABs: harmful algal blooms
MDA: malonaldehyde
NMR: nuclear magnetic resonance
ROS: reactive oxygen species
SOD: superoxide dismutase.
