# Supplementary material for: Algicidal Effects of Prodigiosin on the Harmful Algae Phaeocystis globosa
Source: Front Microbiol. 2016 Apr 26;7:602. doi: 10.3389/fmicb.2016.00602 (PMC4844626; doi:10.3389/fmicb.2016.00602)
Supplement: Supplementary file 1 [file Data_Sheet_1.PDF]

Effectiveness of an anti-algal compound in eliminating an aquatic unicellular harmful algal *Phaeocystis globosa*

Huajun Zhang[1,2], Yun Peng[1], Su Zhang[1], Guanjing Cai[1], Yi Li[1], Xujun Yang[1], Ke Yang[1], Zhangran Chen[1], Jun Zhang[1]， Hui Wang[3], Wei Zheng[1,2]*, Tianling Zheng[1,2]*

AUTHOR ADDRESS:

1. Key Laboratory of the Ministry of Education for Coastal and Wetland Ecosystems, School of Life Sciences, Xiamen University, Xiamen, China

2. School of Marine Sciences, Ningbo University, Ningbo 315211, China

3. Department of biology, college of science, Shantou university. 515063, China

*Corresponding author:

Tianling Zheng : wshwzh@xmu.edu.cn; Wei Zheng: jedi@xmu.edu.cn;

Supplementary Material

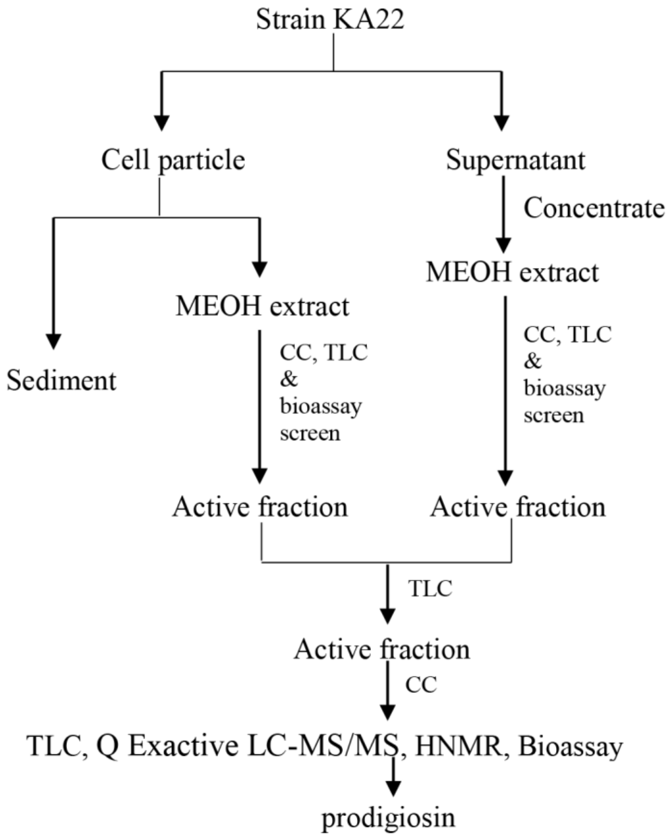

Figure S1. The flow chart for the isolation and identification of the anti-algal compound from strain KA22. (MEOH, methanol; EA, ethyl acetate; CC, column chromatography; TLC, thin layer chromatography)

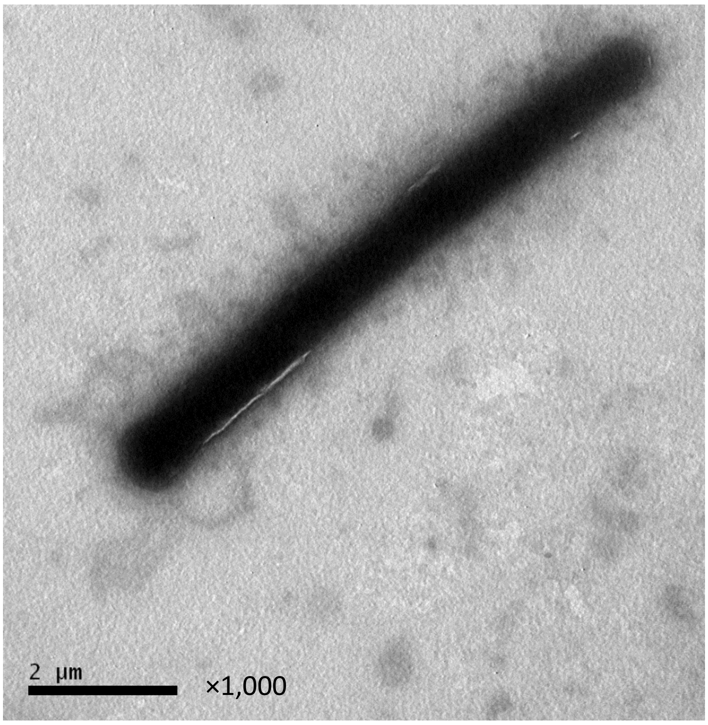

Figure S2. Transmission electron micrographs of bacterial isolate KA22.

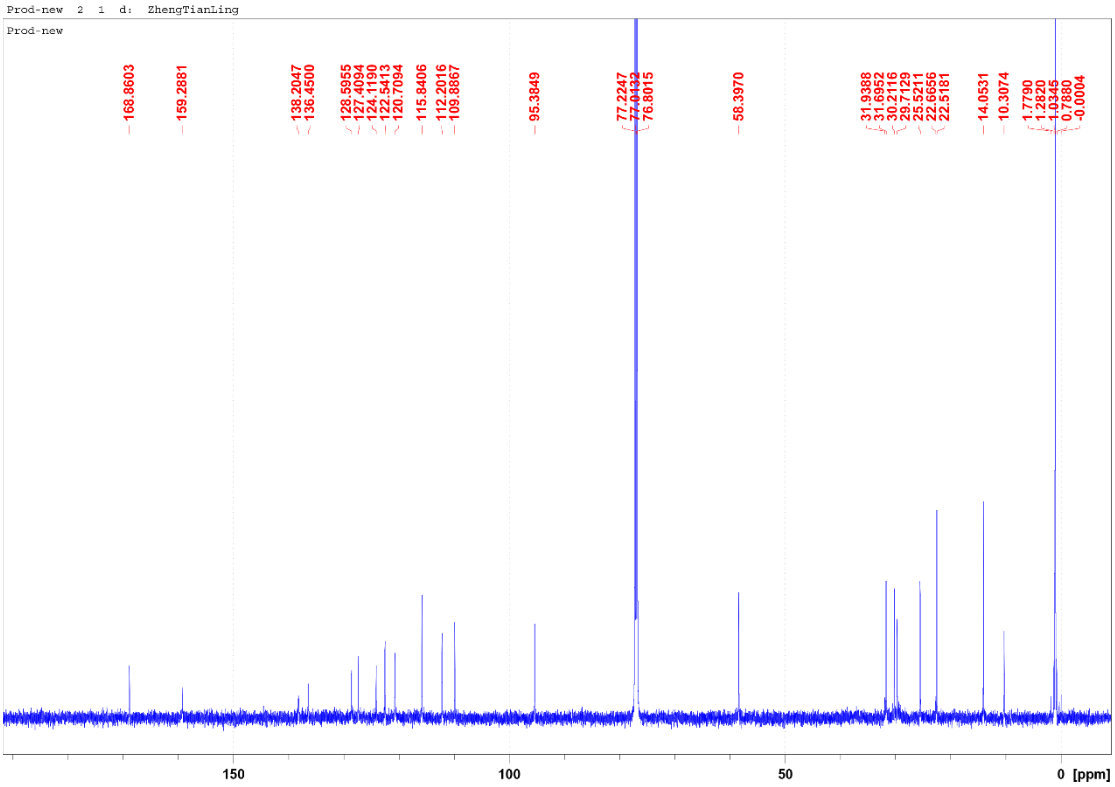

Figure S3. **^{13}C NMR spectrum of prodigiosin in $CDCl_3$.**

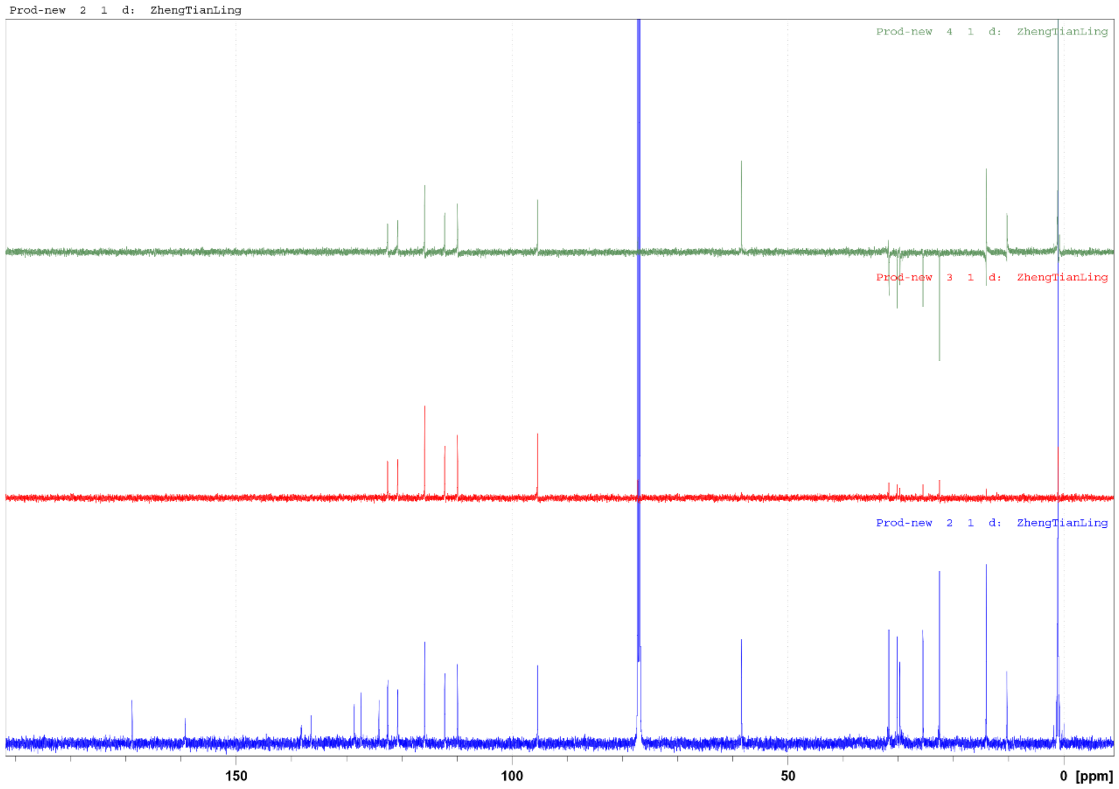

Figure S4. DEPT spectrum of prodigiosin in $CDCl_3$.

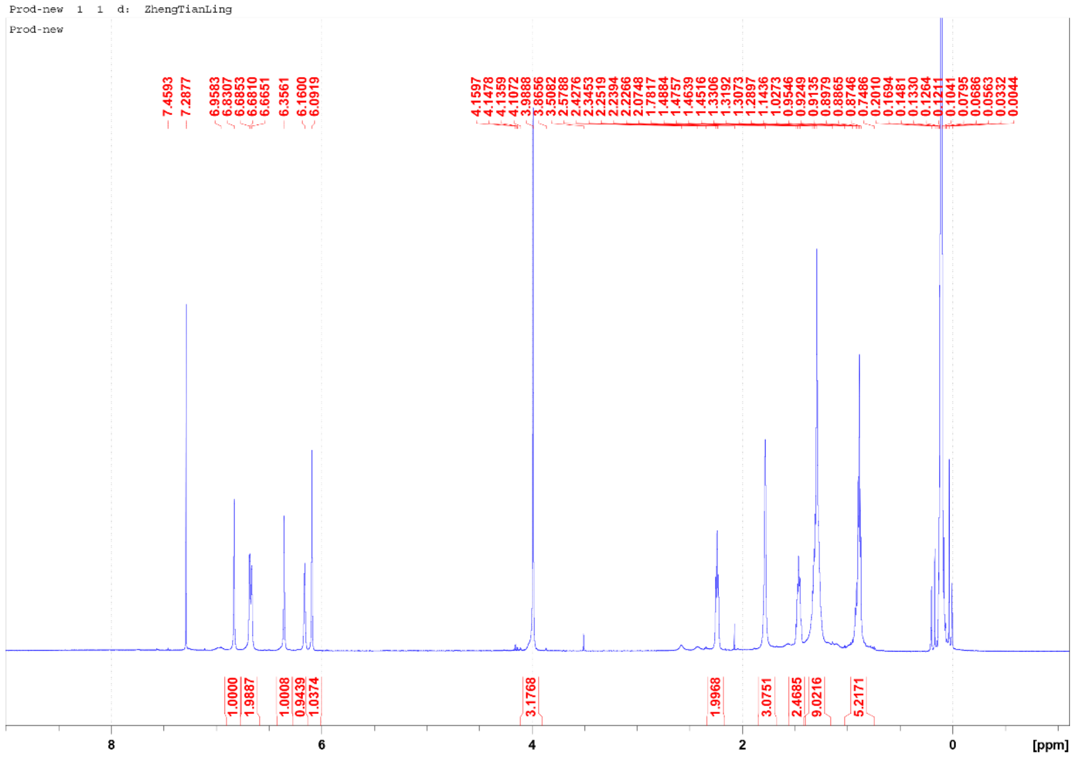

Fiugure S5. ¹H-NMR spectrum of prodigiosin in $CDCl_3$.

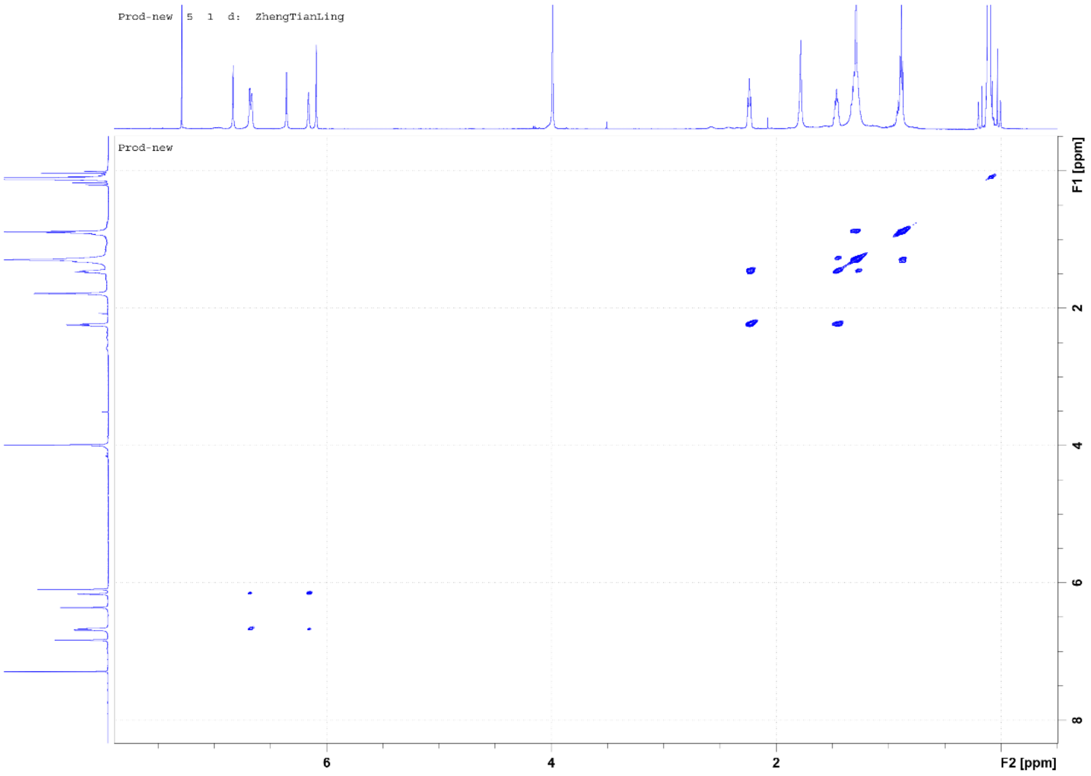

Figure S6. ^1H-^1H COSY spectrum of prodigiosin in $CDCl_3$.

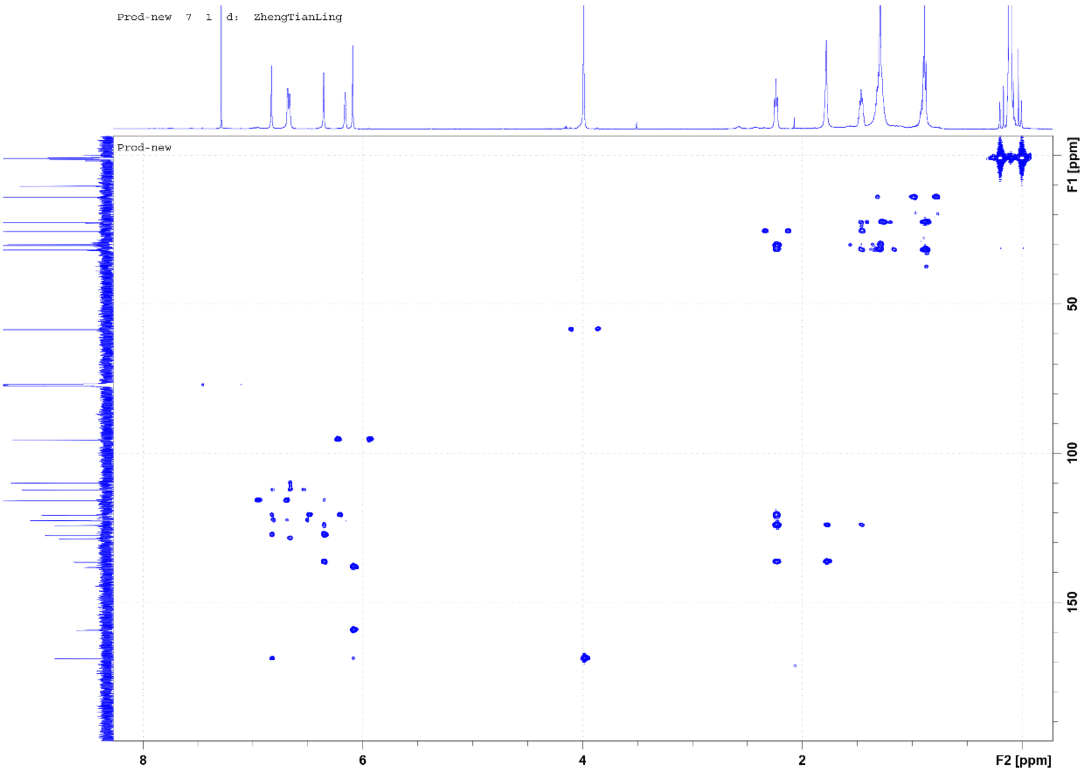

Figure S7. HMBC (heteronuclear multiple-bond correlation) spectrum of prodigiosin in $CDCl_3$.

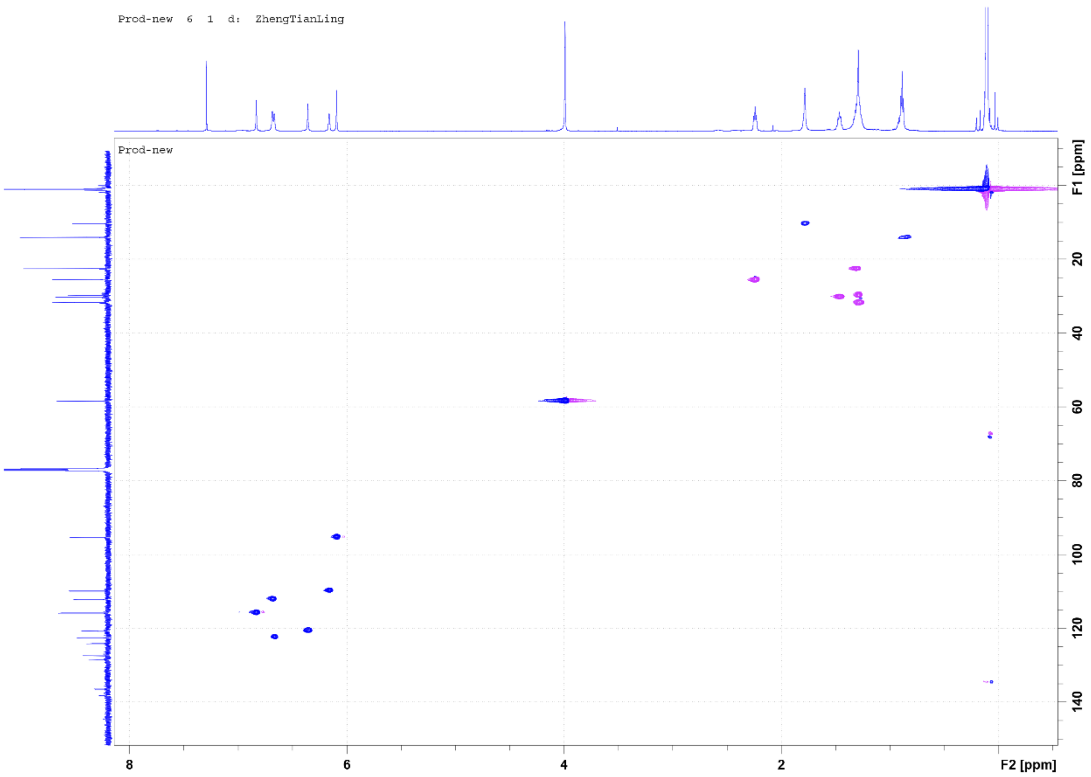

Figure S8. HSQC (heteronuclear singular quantum correlation) spectrum of prodigiosin in $CDCl_3$.
